# Community evolution in patent networks: technological change and network dynamics

**DOI:** 10.1007/s41109-018-0090-3

**Published:** 2018-08-13

**Authors:** Yuan Gao, Zhen Zhu, Raja Kali, Massimo Riccaboni

**Affiliations:** 1 IMT School for Advanced Studies Lucca, Piazza San Francesco 19, Lucca, 55100 Italy; 2 Department of International Business & Economics, University of Greenwich, Park Row, London, SE10 9LS UK; 3 Department of Managerial Economics, Strategy and Innovation, Katholieke Universiteit Leuven, Oude Markt 13, Leuven, 3000 Belgium; 4 Department of Economics, University of Arkansas, University of Arkansas, Fayetteville, 72701 AR USA

**Keywords:** Technological change, Temporal networks, Patent data, Louvain community detection method, Overlapping community mapping

## Abstract

When studying patent data as a way to understand innovation and technological change, the conventional indicators might fall short, and categorizing technologies based on the existing classification systems used by patent authorities could cause inaccuracy and misclassification, as shown in literature. Gao et al. (International Workshop on Complex Networks and their Applications, 2017) have established a method to analyze patent classes of similar technologies as network communities. In this paper, we adopt the stabilized Louvain method for network community detection to improve consistency and stability. Incorporating the overlapping community mapping algorithm, we also develop a new method to identify the central nodes based on the temporal evolution of the network structure and track the changes of communities over time. A case study of Germany’s patent data is used to demonstrate and verify the application of the method and the results. Compared to the non-network metrics and conventional network measures, we offer a heuristic approach with a dynamic view and more stable results.

**Electronic supplementary material:**

The online version of this article (10.1007/s41109-018-0090-3) contains supplementary material, which is available to authorized users.

## Introduction

Patent data has attracted the interest of researchers as a way to measure and understand innovation and technological change, especially with the increased availability of online electronic database and the efforts made by worldwide patent authorities to consolidate and harmonize patent data at international level (Maraut et al. 2008; OECD 2009).

Gao et al. (2017) have introduced an approach to construct networks based on the OECD Triadic Patent Family database (Dernis and Khan 2004), to identify communities and the community cores. The comparison against the International Patent Classification (IPC) system (WIPO 2017a; 2017b) shows that the endogenous communities can provide a more accurate and complete list of potentially associated IPC classes for any given patent class. This association is indicated by being the most consistent nodes in the community containing the given node, as measured by an indicator named coreness. However, that approach was unable to effectively capture the temporal evolution of a community over time due to the difficulty in community tracking.

This paper continues to address this unsolved problem. For community identification, we use an improved Louvain modularity optimization algorithm. To define community cores, we have developed a heuristic approach to detect the central groups of nodes based on the intrinsic characteristics of the temporal networks. As for community tracking, we use a method to find the “best match” based on majority nodes mapping to the reference community. Verification and robustness checks show that our findings are sound and reliable. We also present a case study to demonstrate the real-world implications of our results.

## Background

Since the Sixties patent data has been used by many researchers to measure patent quality, their economic value and possible impact on technological developments and economy (Griliches and Schmookler 1963; Comanor and Scherer 1969; Griliches 1998; Squicciarini et al. 2013; Hausman and Johnston 2014). Most of the well-recognized conventional indicators are straightforward measures, such as the number of patent applications and publications, time needed from filing to grant (grant lag), number of different technology classification codes involved (patent scope), forward and backward citation counts, etc. Such indicators may be used to track technological changes and innovation, but when considered alone, will fall short due to their simplicity and lack of context, resulting in bias and sometimes contradicting conclusions (Benner and Waldfogel 2008; Dang and Motohashi 2015; Hall et al. 2001; Hall and et al. 2005;Harhoff et al. 2003).

In the light of this, we carried out the previous research (Gao et al. 2017*) to study patent data from a network perspective (*Acemoglu et al. 2016*), which lays the foundation for the motivation of this paper. More specifically, two types of networks are constructed based on how individual patents grouped into the same family, and how patents in different families cite each other. In both networks, the nodes are the 4-digit subclass level IPC codes following WIPO’s IPC scheme of 2016 (*WIPO 2016). This paper focuses on the former type, the family cohort network, in which any two of the total of 639 nodes are connected when they are both found in patents of the same patent family. The more times two subclasses nodes are found to share the same family, the more intense they are linked in the network. Based on this construction mechanism, a community of closely connected nodes indicates that the represented technological fields are more likely to be found in the same inventions. For example, pharmaceutical products in IPC class A61 and enzymology or microbiology in class C12 frequently co-occur in patent families and they are found to be in the same network community.

Application inventions usually involve more than one technology field. A car, for example, consists of many parts serving different functions. Innovations in molecular material science could stimulate the birth of a new type of tire, or a more efficient type of fuel, which then brings a new design of engines involving mechanical and electronic innovations. Along the technological trajectories there are many cases like this. To find out how an established community of technological changes over time, splitting up and merging with other technologies, is not only interesting in the retrospective observation of technological development trends, but also helps in understanding the interactions between science and technology and policy making, market drives and other socio-economic factors.

## Data

The dataset used for the analysis is retrieved from the February, 2016 edition OECD patent database (OECD 2017*). In addition, ISO country codes from the OECD REGPAT database (*Maraut et al. 2008) are used to sort out patent families by country.

In this paper, a patent family from a country is defined as a family containing at least one patent of which at least one applicant is from that country. The applicant’s country is used instead of the inventor’s country because the applicants designate the owners or party in control of the invention, mostly firms (OECD 2009). Therefore it reflects the innovative performance of the given country’s firms, while the inventor’s country is usually the inventor’s professional address.

The REGPAT database is most reliable for OECD and EU countries since it is based on two sources: patent applications to the European Patent Office (EPO) and filed under the Patent Co-operation Treaty (PCT) from 1977 to 2013. We chose to focus on Germany for our case study and we use the data from year 1980–2013 for more consistent data quality. Germany has the largest number of patent applications among all the EU countries, and ranks third for patent production among the OECD countries.

## Methodology

The analysis mainly consists of three parts, to be described in the following paragraphs: community identification, central nodes identification and community tracking over time.

### Community identification

In our previous study (Gao et al. 2017*), we used the Lumped Markov Chain method proposed by Carlo Piccardi (*2011) to detect clusters in networks. This method produces satisfying results for a single static network with sufficiently strong clustering structure. However, for our purpose to analyze the temporal evolution of a network, essentially a network in multiple time slices, this method would treat each time slice as a separate network without connection to each other, which is not appropriate for the continuous technological development issue of interest. Also, the marginal results observed show that the detected community structure is very sensitive to the input network. In other words, although the network is not supposed to have dramatic change from one snapshot in time to the next, a small change could cause significant transformation in the resulting communities.

To better capture the network’s temporal properties and overcome the instability, we use a modification of the Louvain modularity optimization method for community detection. This modification, namely the Stabilized Louvain Method, proposed by Aynaud and Guillaume (2010*), has been proved to achieve more stable results in tracing communities over time. The Louvain method finds the community structure with maximum modularity by looking for modularity gain through iterations (*Blondel et al. 2008). The modification, essentially, is to change the initial partition of the network at time *t* to the detected partition at time *t-1*, thus the initial partition is constrained to take into account the communities found at the previous time steps, making it possible to identify the real trends.

The algorithm implementation is based on the Python module using NetworkX for community detection (Aynaud 2009). We split up the database by the earliest priority year of patent family, and execute the algorithm for each year, using the detected network partition as the initial partition for the next year.

### Central nodes identification

There are many different ways to define centrality within a community and/or a network, from the classic definitions by degree, betweenness, closeness, Eigenvector, PageRank, etc, to many customized concepts in empirical and theoretical researches (Freeman 1978*;*Wasserman and Faust 1994*;*Valente et al. 2008*). For example, in our previous work (*Gao et al. 2017) “Coreness” has been defined as a measure of weighted centrality, based on the probability to be present in the community and the intra-community centrality of each node. However, similar to community detection, centrality measures are not designed for temporal evolving networks and the adoption of one metric out of the others is usually an ad-hoc choice.

A more heuristic concept of cores, as defined by Seifi and colleagues, is certain sets of nodes that different community detection algorithms or multiple execution of a non-deterministic algorithm would agree on (Seifi et al. 2013*). They summarized that for a static network, there are two types of algorithms to identify such sets of nodes: by adding perturbations to the network, and by changing the initial configuration. In the first type, small perturbations such as removing a fraction of links and putting them back on random pairs of nodes, are used to create slightly different networks from the original and produce different partitioning results for comparison and finding of the consensus communities. However, for a network that changes over time, such perturbations naturally exist in each time slice. In fact, they are the temporal changes to be discovered. Therefore, the latter type is more appropriate. Wang and Fleury experimented with the overlapping community technique in a series of works (*Wang 2012*;*Wang and Fleury 2010*;*^2013^*). Our method is similar to the concept of Wang and Fleury’s fuzzy detection method to identify modular overlaps, which are groups of nodes or sub-communities shared by several communities (*Wang 2012), with a different implementation.

We describe the overlapping community mapping algorithm and the central nodes identification methods in a 4-step procedure:

**I.** Given the network partition *P* in the reference time slice *t*, identify a community *C* (*C* ∈*P*). *C* is the target community of interest to be mapped to in the following time slices.

*P* is obtained using the Stabilized Louvain Method described in the previous subsection. For a network with the total set of nodes *E*, *P* = {*C*_1_,*C*_2_,...*C*_*k*_}, where:

$$\bigcup_{i} {C_{i}} = {E}, \\ i \neq j \Rightarrow {C_{i}} \cap {C_{j}} = 0 $$

**II.** In the network with partition *P’* of a following time slice *t’*, find the community with the most nodes in *C*, and that is the mapped community *C’* of *C*. The change of *C* from *t* to *t’* is considered the change between *C* and *C’*. This step can be illustrated by the pseudo codes in Algorithm 1.


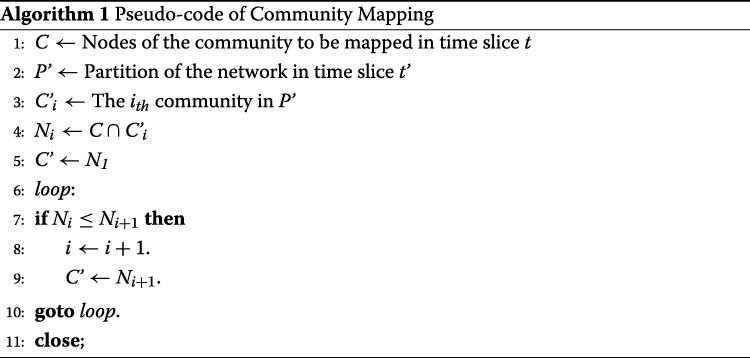


**III.** Based on the communities detected in the previous step, take any node *k*, find the community *C*_0_ it belongs to in the initial year *T*_0_ in a certain time window of *n* years, and use the mapping algorithm to track *C*_0_ in the following years within the time window.

**VI.** The more significant this node *k* is, the more likely it is to be found in the mapped communities. Each node will have a number *W*_*k*_ (*W*_*k*_ ≤*n*) of how many times it is included in the mapped communities throughout the time window. The group of nodes with the largest *W*_*k*_ will become the central sets in this time window. Step III and VI can be illustrated by the pseudo codes in Algorithm 2.


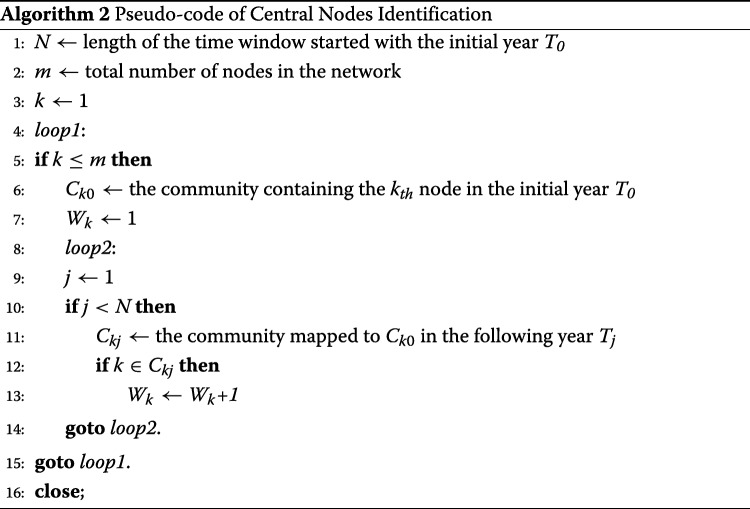


This method uses the intrinsic temporal dynamics of the network to find the central nodes. It is intuitive and heuristic, independent of arbitrary ad-hoc choices of measures. The configuration of the initial year and the length of time window could significantly affect the results. Therefore, robustness checks using different lengths of rolling time windows are necessary to verify stability.

### Community tracking over time

After sets of central nodes are identified, it is then possible to track the community containing them through the years. The tracking method is the same as the mapping algorithm described above. Visualization helps to show that the central nodes are the persistent “cores” of the community under tracking whereas the “peripheral” nodes reflect the changes over time.

## Case study

Using the 3-step method described above, we perform a case study using data of patent families with Germany as the applicant’s country. As the largest economy of the EU, Germany also ranks top among all the EU countries in terms of IP filings, including patent applications. Data from the World Intellectual Property Organization (WIPO) (statistics database W 2017) shows that 176,693 patents have been filed to Germany’s patent office in 2016 from residents and abroad, more than twice of 71,276 from France, the second place in EU. WIPO’s statistics also reports that the top 5 fields of technology associated with patent applications are transport; electrical machinery, apparatus; mechanical elements; engines, pumps, turbines; and measurement.

***Analysis configuration***

In our method, there are several adjustable parameters:

- *Community Detection Resolution*. In the first step, the Louvain method allows for different resolution settings, an implementation of the idea raised by Lambiotte and colleagues that time plays the role of an intrinsic parameter to uncover community structures at different resolutions (Lambiotte et al. 2008). To test the influence of resolution, we run community detection using different resolutions ranging from 0.5 to 2.
- *Overlapping Community Reference*. In the second step, there are two ways to choose the reference year: For any year *T*_*t*_ of the non-initial years in the time window, always refer to the initial year *T*_0_, or refer to the previous year *T*_*t*−1_. The latter would mediate the dependency of the initial year. We have applied both types of time referencing and compared the results.
- *Time Window Setting*. As mentioned in the previous section, the initial year’s network partition is used as the reference for the following years’ community mapping. The time window length is important for two reasons: first, depending on the pace of technology development and potential events driving the changes, the period of time that the initial year would remain valid as the reference varies; and second, longer time windows would require a node to be more “central” to appear at all time or most of the time, and therefore would result in smaller sets of central nodes than shorter time windows. To address these concerns, we used different rolling window settings, including 5 or 10-year time windows with the initial year rolling from year to year (for example, 1980–1989, 1981–1990, …), and 5 or 10-year time windows with the initial year rolling 5 years apart (for example, 1980–1989, 1985–1994, …).

## Results

**Community detection - quantities and sizes:** For community detection, we apply the stabilized Louvain method on the entire time range from 1980 to 2013 because technological development is continuous through all the years.

**Fig. 1:**
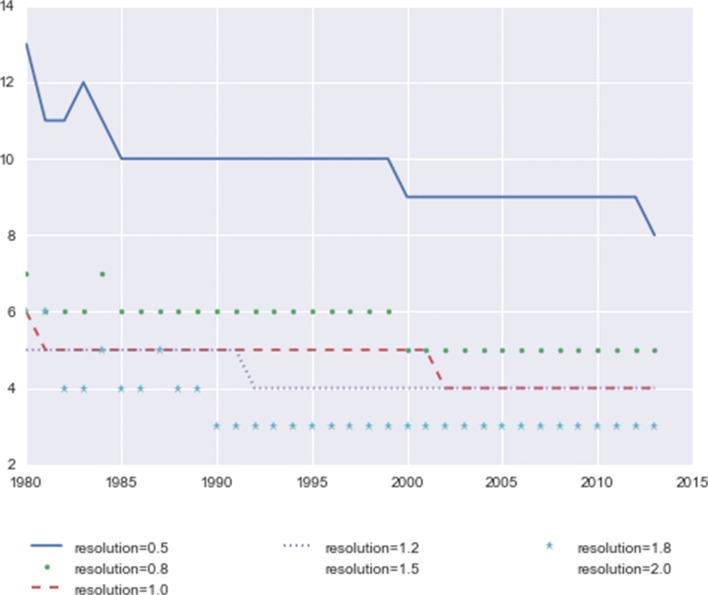
Number of communities at different resolutions. The x-axis indicates years from 1980 to 2013, and the y-axis indicates number of communities

We first check the number of communities detected at different resolution levels. As each node represents a subclass in the IPC scheme, not all of them would appear in every year’s patent applications. In addition, some patent families contain just a single subclass. Such cases would result in “orphan communities”, communities that have only one node without connection to any other nodes. There are also some very small communities with 2 or 3 nodes. Additional file 1: Figure S8 shows the community structure of selected years with resolution set to 1.0, including all the small communities and orphans with nodes layout using Fruchterman-Reingold force-directed algorithm (Hagberg et al. 2008*;*Fruchterman and Reingold 1991). Each sample year has an average of 151 orphan nodes plus 7 nodes in small communities with no more than 5 nodes. So many isolated nodes and small communities will cause too much noise in the analysis. To focus on the meaningful clusters, we have excluded all the communities with 5 nodes or less from the detected partitions. Quantities of the remaining communities are shown in Fig. 1.

Contrary to the common wisdom that higher resolutions correspond to finer, and therefore more partitions, the figure shows that after excluding the very small communities, the lowest resolution 0.5 has the most communities in all the years, and resolutions 1.8 and 2.0 have the fewest. Figure 1 also shows that the community numbers generally have a decreasing trend over the years. This is due to the mechanism of the stabilized algorithm where each year’s initial partition builds on the previous year. With the enhanced stability, it becomes easier to identify clusters with time. It is noteworthy that the decrease of number of non-tiny communities over time does not indicate the breakdown of weakly connected communities, but rather community merging, including the situation where a community splits into 2 or more smaller parts which merge into other large communities.

Likewise, one should be aware that the disappearance of a portion of nodes in a community does not mean such nodes abruptly disconnect from the central nodes of the community. They are most likely still connected, but have become more closely connected with another set of central nodes, or are replaced by other nodes that are closer to the original central nodes. The methodology of cluster identification involves such “competition” at all times.

**Fig. 2:**
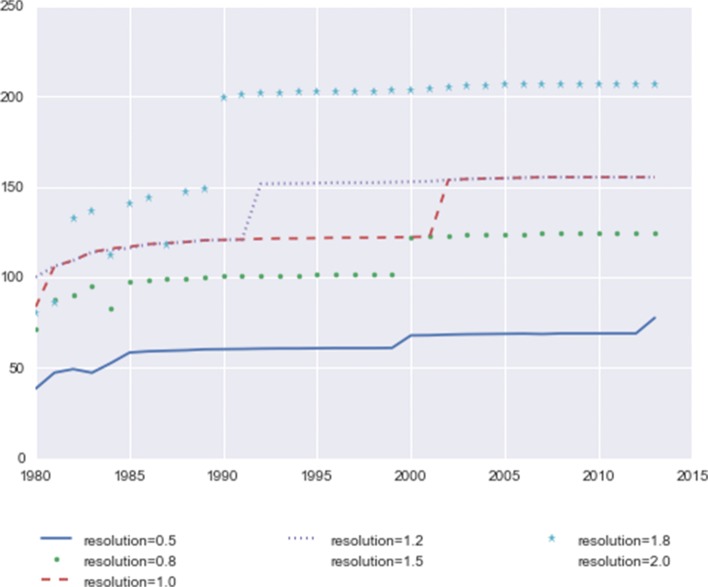
Average community size at different resolutions. The x-axis indicates years from 1980 to 2013, and the y-axis indicates number of nodes in the community

We also check the community sizes. Figure 2 shows the average number of nodes in community for all the years at different resolutions. Overall, the community size increases with resolution, and from the earlier years to the more recent years.

The first-step results show that although the algorithm detects more, finer communities under higher resolutions, a lot of them are very small communities. As a result, at the higher resolutions the community size distribution tends to be more polarized, with fewer but more aggregated communities, and more tiny communities than at the lower resolutions.

**Central nodes - occurring rate:** Similarly, different resolutions would result in different sets of central nodes. We define an indicator named “occurring rate” as the number of occurrence of each node in the mapped communities, divided by the total number of years in the time window. For a year-to-year rolling time window setting, the average occurring rate of all the nodes over a certain time window is calculated as

1 $$ O_{r} = \frac{\sum_{t=1}^{34-N+1}\left(\frac{\sum_{i=1}^{m}\left(\frac{n_{{ir}}}{N}\right)}{m}\right)}{34-N+1}, r\in\{0.2, 0.5, 1.0, 1.2, 1.5, 1.8, 2.0\}, m\leq 639  $$

where *m* is the total number of nodes in year *t* after excluding those very small communities with less than 5 nodes; *n*_*ir*_ is the occurrence of the *i*_*th*_ node at resolution *r* in each time window during the community mapping process including the initial year; and *N* is the length of the time window.

**Fig. 3:**
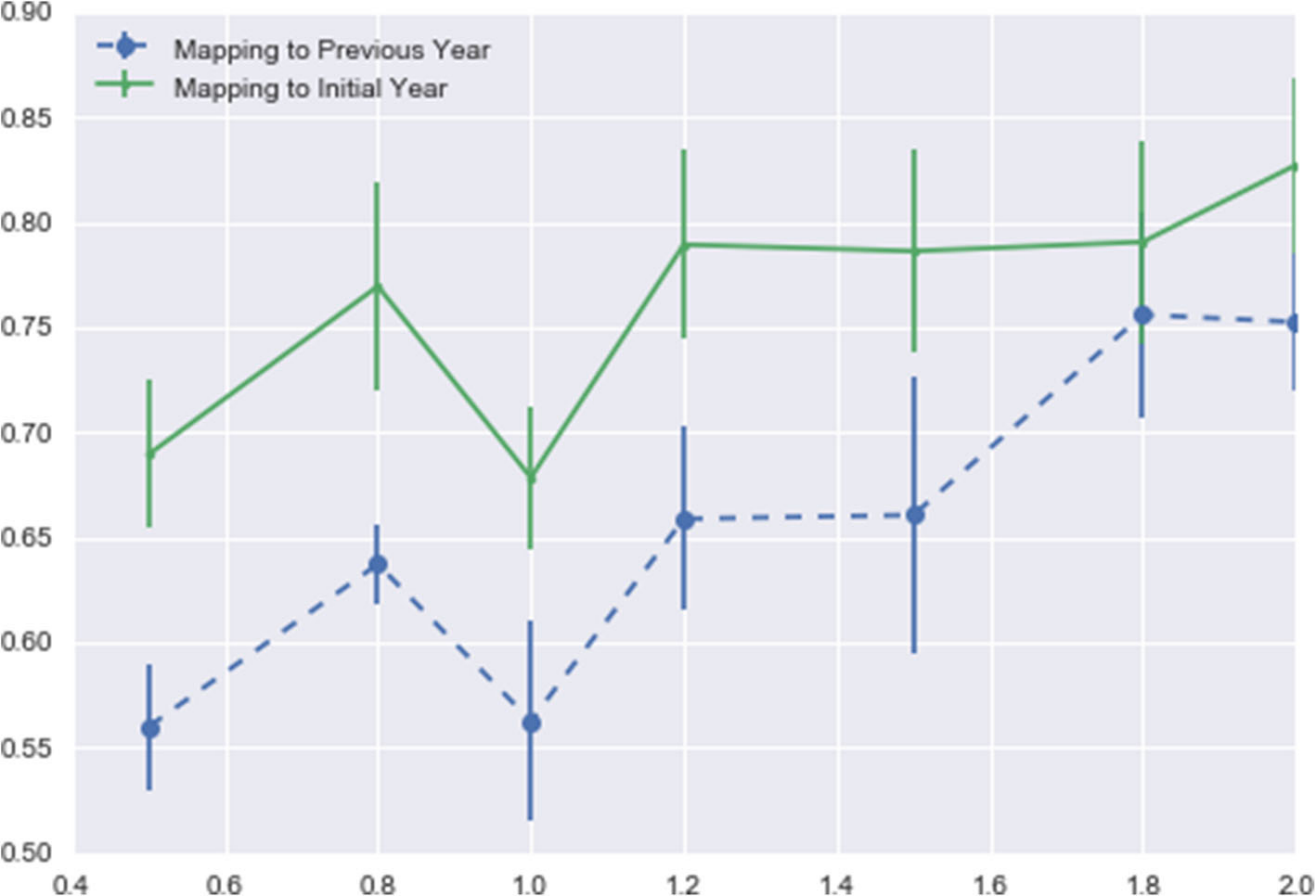
Occurring rate statistics at different resolutions. The mean value and standard deviation of the occurring rate over all the nodes, using the 10-year time window rolling from year to year, including 25 time windows with initial years from 1980 to 2004. The x-axis indicates various resolution values, and the y-axis is the scale of mean values. Results from two mapping methods are shown in this figure

We use the configuration of 10-year windows rolling from year to year to demonstrate this result. When *N* is equal to 10 in Eq. 1, the calculated mean values and standard deviations of the occurring rates at various resolutions are shown in Fig. 3. Using both mapping algorithms, the lowest average occurring rates are at resolution 1.0.

While there is no benchmark for the absolutely ground truth to determine which resolution is the “best”, for our analysis purpose there are some preferred qualities: lower average occurring rates are more desirable because such community structures can better reflect the changes over time: Figs. 1 and 2 show that the higher resolutions generate fewer and larger communities, which indicates that the community sizes tend to polarize at higher resolutions, with fewer large communities and more tiny communities, or even disconnected single-node communities.

At lower resolutions, the number of communities larger than five increases, which might also bring more instability (the number of distinct communities decreases from 13 to 8 at resolution 0.5). Therefore, we choose resolution 1.0 as the setting for the next step, to identify communities and track them over time.

The statistical behavior shown above under different resolutions is related to the problem known as “resolution limit” (Fortunato and Barthelemy 2007*), that the modularity optimization method may fail to identify communities smaller than a certain scale. Lambiotte and colleagues have also verified in their framework that partitions beyond a certain resolution limit are obtained at small time where the optimal partition is the finest (*Lambiotte et al. 2008).

**Fig. 4:**
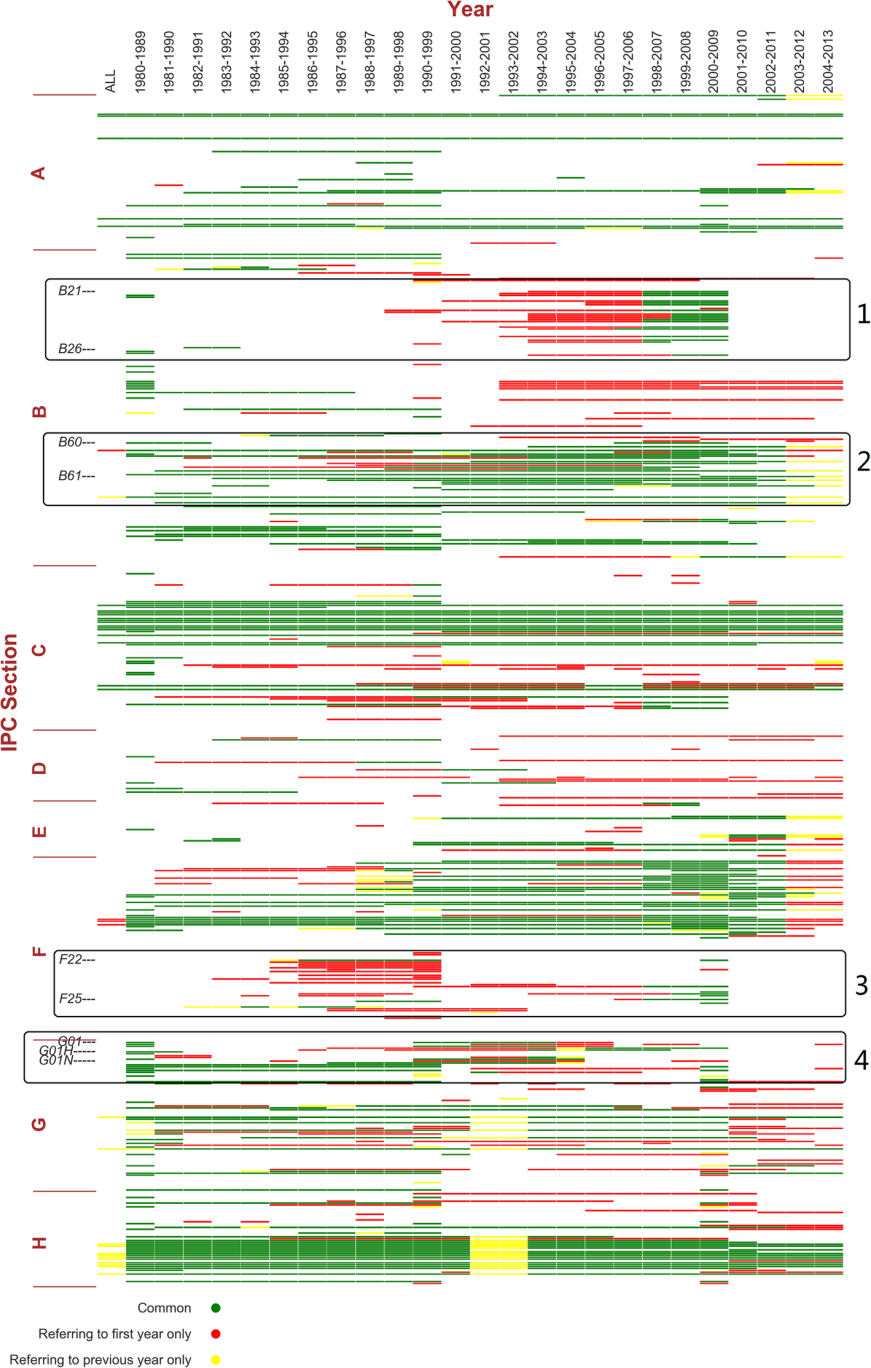
Central nodes of 10-year time window rolling from year to year. The x-axis indicates time windows from 1980–1989 to 2004–2013, except for the first column labeled “ALL”, which is the all-year condition. The y-axis indicates the nodes, i.e. IPC subclasses, ordered in IPC index. Colored blocks indicate central nodes in a time window using at least one referencing method: Green represents central nodes both methods have in common, red for those central only by referring to the initial year, and yellow for those central only by referring to the previous year

**Central nodes at resolution 1.0:** Using the overlapping algorithm, at resolution 1.0, we select different time window configurations to identify the central nodes, each with the two referencing methods described above. Figure 4 shows the central nodes plotting under the 10-year time window setting, rolling from year to year. The threshold of the central nodes is set to be the length of the time windows (34 for the all-year setting and 10 for the rolling windows). That is, only the most persistent nodes with an occurring rate of one within the time window are colored in the figure. So under the all-year setting there are fewer central nodes. If a node is central using both referencing methods (colored green), it is more likely that the initial community has not gone through significant reshuffling. If it is only central when referring to the initial year (colored red), then in at least one of the following years in the time window, the initial community has probably experienced some changes that are not in a consistent direction. For example, when merging and then splitting, by referring to the previous year a node might be left out in the minority part of the merged community. If a node is central only when referring to the previous year, it is likely that it has just drift away from the initial community during accumulated changes. For some nodes, they would become red first, and then turn to green. This means the changes have stabilized.

Figure 4 shows several noteworthy trends, highlighted as framed areas 1–4. However, at this moment it is too soon to relate these signals with real-world facts since it is not yet clear how central nodes are grouped into different communities. At this stage, the visualization provides a guidance for the potential trends to take a closer look at. Overall, it also shows the most persistent central nodes, such as IPC Class C07-C08 (organic chemistry and organic macromolecular compounds), and H03-H04 (electric circuitry and electric communication technique).

**Community tracking:** At this step, any chosen community in the initial year can be tracked to analyze its changes over time. We use two examples to illustrate our approach. Since the endogenous communities do not have meaningful names, we refer to them by one of the representative central nodes they contain in year 1980’s partition: B01D, defined in IPC as “separation in physical or chemical processes”; and B60R, “vehicles, vehicle fittings, or vehicle parts” not provided for in other categories under class B60, “vehicles in general”.

**Fig. 5:**
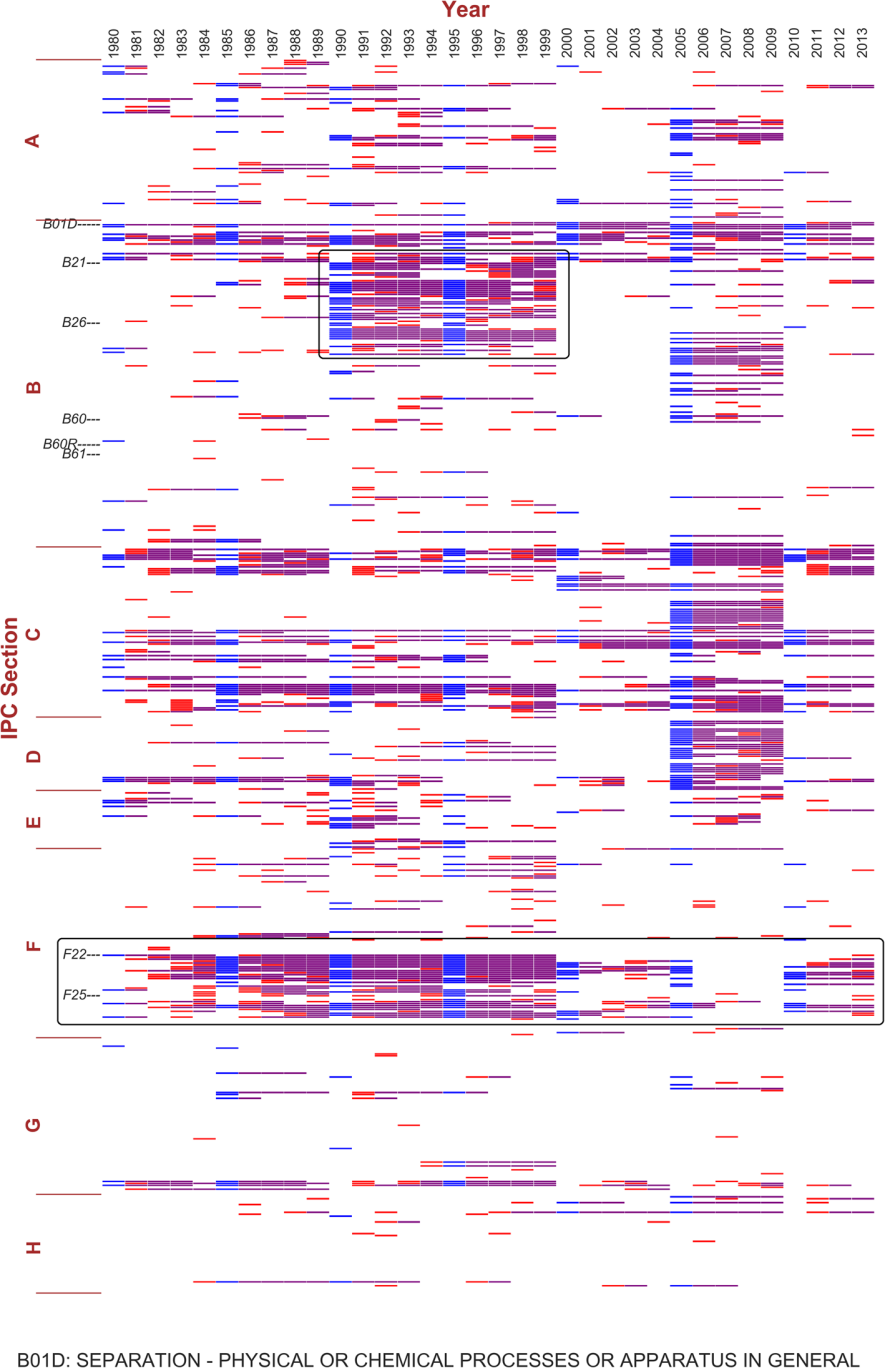
Tracking community B01D in consecutive 5-year time windows, mapping to the previous year. The x-axis indicates years from 1980 to 2013, and the y-axis indicates the nodes, i.e. IPC subclasses, ordered in IPC index. The community mapping is based on consecutive 5-year windows. In each time window, the initial year’s community containing the central node set represented by B01D is shown in blue. In the rest 4 years, colored nodes represent the mapping communities: red indicates the node does not exist in the reference community (community of the previous year), and purple indicates the overlapping part

**Fig. 6:**
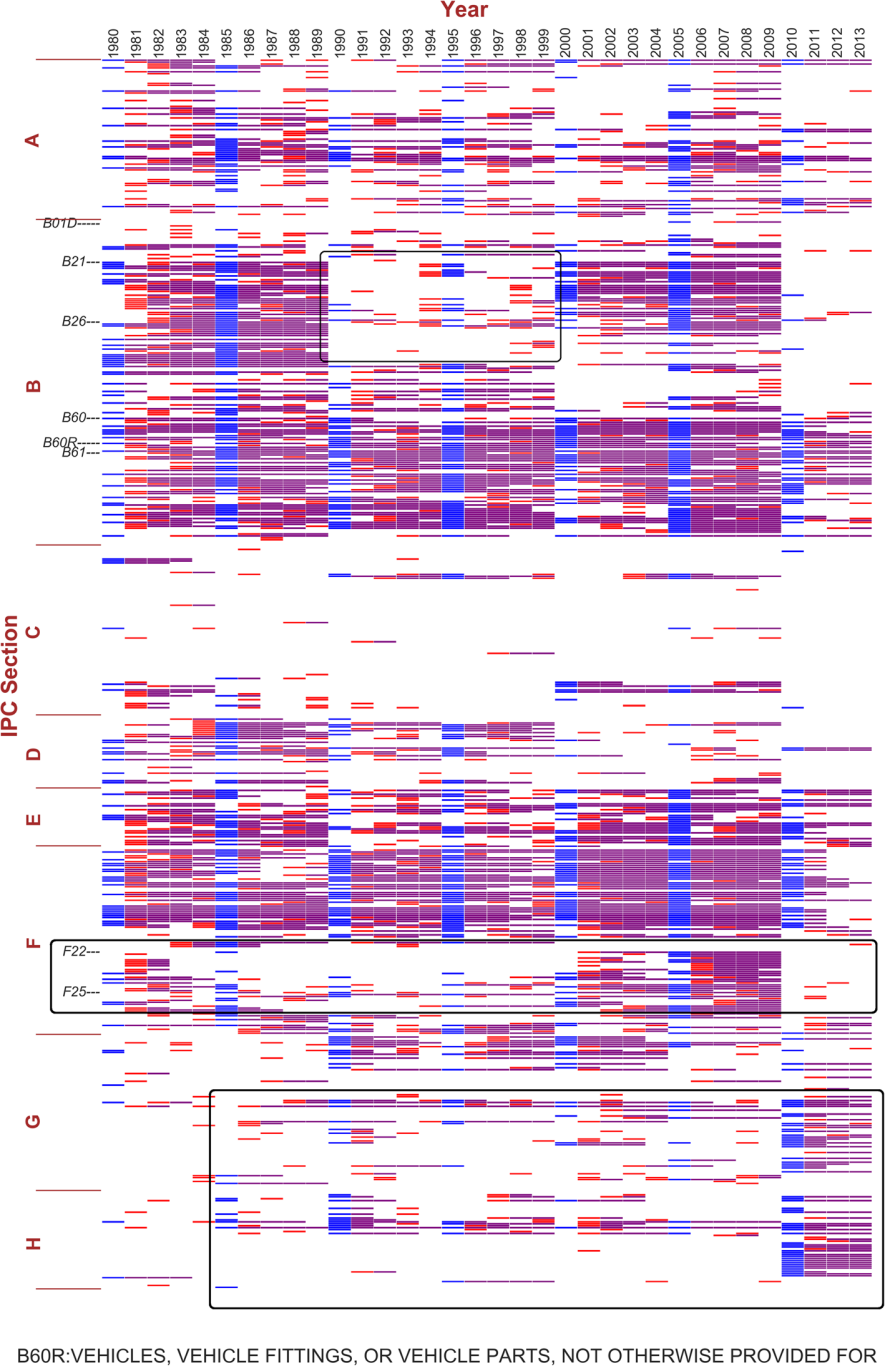
Tracking community B60R in consecutive 5-year time windows, mapping to the previous year. The x-axis indicates years from 1980 to 2013, and the y-axis indicates the nodes, i.e. IPC subclasses, ordered in IPC index. The community mapping is based on consecutive 5-year windows. In each time window, the initial year’s community containing the central node set represented by B60R is shown in blue. In the rest 4 years, colored nodes represent the mapping communities: red indicates the node does not exist in the reference community (community of the previous year), and purple indicates the overlapping part

Figures 5 and 6 show the results of tracking the two communities above, respectively. Both communities cover multiple IPC sections, as discussed by Gao et al. (2017). The two figures show that the consistently overlapping parts of the two communities are different most of the time. B01D’s community mainly consists of various physical or chemical processes treating materials and tooling (classes B01-B06), artificial materials from glass to cement and ceramics (C02-C03), petrol and gas industries (C10), and metallurgy and metal surface treatment (C21-C23). Such a composition suggests the application of physical or chemical processing techniques in the inventions of certain industries. For B60R, its community covers the majority of Section B, E and classes F01-F17, a combination of machinery, mechanical engineering, vehicles and transportation, building and construction. Relating to Fig. 4, the central nodes of these two communities contribute to a majority of the central nodes, including the framed areas 1 and 2. This is consistent with the WIPO statistics about Germany’s top technology fields of patent applications (for the complete IPC definitions, please refer to WIPO’s IPC Scheme (WIPO 2016)).

Next, we focus on the major differences between the two figures. From 1990 to 1999, B21-B30 “moves” from Fig. 6 to Fig. 5. Those subclasses focus on technologies related to metal working, machine tools, and hand tools, which are likely to be applied in both communities. The temporary “move” turns back after 1999. This is an example of marginal clustering. Another similar case is the “move” of classes F22-F25 from Figs. 5 to 6 from 2000 to 2009. This part represents technologies related to combustion process, heating and refrigeration. After robustness check, the “moves” still exist. This indicates that instead of an artifact due to time window configuration, the “moving” technologies are closely connected to both communities and the network clustering algorithm captures the changes in the relative connectivity. These two “moving” parts also provide an explanation to the framed areas 1 and 3 in Fig. 4: the temporary community switches may result in the rise and fall of a set of central nodes in the following or preceding rolling time windows.

In Fig. 6, we should also notice the spread to Section G and H starting from the 1990s. This is a consistent trend, getting stronger in the last 4 years. Compared to Fig. 5 which also covers a part of Section G, the community containing B60R incorporates more technologies in digital computer (class G06), electric devices and power supply and distribution (class H01 and H02). This observation is in line with WIPO’s report of electrical machinery as the second top technology field of patent applications (statistics database W 2017).

## Discussion

**Technological change in Germany’s automotive industry:** To make sense of data analysis findings based on real-world technological trends is always difficult. In most empirical analysis, reliable methods and domain knowledge in the industry are both essential.

In Fig. 6, the technology community containing B60R takes up more than half of Germany’s patent filing activities, with the most persistent parts being IPC classes B60-B67, Section E, and F1-F16. These technologies can be considered as the mainstream of this community: vehicles and transportation, building and construction, machine and engines (for the details of these IPC schemes, please refer to Table 1).

**Table 1:** IPC schemes of the persistent technologies in community containing B60R

Section	Class	Scheme
B	B60	VEHICLES IN GENERAL
	B61	RAILWAYS
	B62	LAND VEHICLES FOR TRAVELLING OTHERWISE THAN ON RAILS
	B63	SHIPS OR OTHER WATERBORNE VESSELS; RELATED EQUIPMENT
	B64	AIRCRAFT; AVIATION; COSMONAUTICS
	B65	CONVEYING; PACKING; STORING; HANDLING THIN OR FILAMENTARY MATERIAL
	B66	HOISTING; LIFTING; HAULING
	B67	OPENING OR CLOSING BOTTLES, JARS OR SIMILAR CONTAINERS; LIQUID HANDLING
E	E01	CONSTRUCTION OF ROADS, RAILWAYS, OR BRIDGES
	E02	HYDRAULIC ENGINEERING; FOUNDATIONS; SOIL-SHIFTING
	E03	WATER SUPPLY; SEWERAGE
	E04	BUILDING
	E05	LOCKS; KEYS; WINDOW OR DOOR FITTINGS; SAFES
	E06	DOORS, WINDOWS, SHUTTERS, OR ROLLER BLINDS, IN GENERAL; LADDERS
	E21	EARTH OR ROCK DRILLING; MINING
	E99	SUBJECT MATTER NOT OTHERWISE PROVIDED FOR IN THIS SECTION
F	F01	MACHINES OR ENGINES IN GENERAL; ENGINE PLANTS IN GENERAL; STEAM ENGINES
	F02	COMBUSTION ENGINES; HOT-GAS OR COMBUSTION-PRODUCT ENGINE PLANTS
	F03	MACHINES OR ENGINES FOR LIQUIDS; WIND, SPRING, OR WEIGHT MOTORS; PRODUCING
		MECHANICAL POWER OR A REACTIVE PROPULSIVE THRUST, NOT OTHERWISE PROVIDED FOR
	F04	POSITIVE-DISPLACEMENT MACHINES FOR LIQUIDS; PUMPS FOR LIQUIDS OR ELASTIC FLUIDS
	F15	FLUID-PRESSURE ACTUATORS; HYDRAULICS OR PNEUMATICS IN GENERAL
	F16	ENGINEERING ELEMENTS OR UNITS; GENERAL MEASURES FOR PRODUCING AND MAINTAINING
		EFFECTIVE FUNCTIONING OF MACHINES OR INSTALLATIONS; THERMAL INSULATION IN GENERAL
	F17	STORING OR DISTRIBUTING GASES OR LIQUIDS

Clear changing trends can also be observed. Aside from the marginal “moves” like B21-B23 as discussed above, we focus on the more consistent trends, such as the increasing involvement of Section G and H, specifically, classes G01, G05, G06, H01 and H02, shown in the bottom framed area in Fig. 6. These are the technologies related to measuring and testing, controlling and regulating, computing, electric elements, and electric power. This trend started from 2000, and became significantly stronger since 2010 (for the relevant IPC schemes of the classes and the subordinate central subclasses, please refer to Table 2).

**Table 2:** IPC schemes of the central nodes in Section G and H in community containing B60R

Code	Scheme
G01	Measuring; Testing
G01F	MEASURING VOLUME, VOLUME FLOW, MASS FLOW, OR LIQUID LEVEL; METERING BY VOLUME
G01G	WEIGHING
G01H	MEASUREMENT OF MECHANICAL VIBRATIONS OR ULTRASONIC, SONIC OR INFRASONIC WAVES
G01L	MEASURING FORCE, STRESS, TORQUE, WORK, MECHANICAL POWER, MECHANICAL EFFICIENCY, OR FLUID
	PRESSURE
G01M	TESTING STATIC OR DYNAMIC BALANCE OF MACHINES OR STRUCTURES; TESTING OF STRUCTURES OR
	APPARATUS, NOT OTHERWISE PROVIDED FOR
G01P	MEASURING LINEAR OR ANGULAR SPEED, ACCELERATION, DECELERATION OR SHOCK; INDICATING
	PRESENCE OR ABSENCE OF MOVEMENT; INDICATING DIRECTION OF MOVEMENT
G01W	METEOROLOGY
G05	Controlling; Regulating
G05B	CONTROL OR REGULATING SYSTEMS IN GENERAL; FUNCTIONAL ELEMENTS OF SUCH SYSTEMS;
	MONITORING OR TESTING ARRANGEMENTS FOR SUCH SYSTEMS OR ELEMENTS
G05D	SYSTEMS FOR CONTROLLING OR REGULATING NON-ELECTRIC VARIABLES
G05G	CONTROL DEVICES OR SYSTEMS INSOFAR AS CHARACTERISED BY MECHANICAL FEATURES ONLY
G06	Computing; Calculating;
G06M	COUNTING MECHANISMS; COUNTING OF OBJECTS NOT OTHERWISE PROVIDED FOR
G08	Signalling
G08G	TRAFFIC CONTROL SYSTEMS
G10	Musical instruments; Acoustics
G10K	SOUND-PRODUCING DEVICES; METHODS OR DEVICES FOR PROTECTING AGAINST, OR FOR DAMPING,
	NOISE OR OTHER ACOUSTIC WAVES IN GENERAL
H01	Basic electric elements
H01H	ELECTRIC SWITCHES; RELAYS; SELECTORS; EMERGENCY PROTECTIVE DEVICES
H02	Generation, conversion, or distribution of electric power
H02G	INSTALLATION OF ELECTRIC CABLES OR LINES, OR OF COMBINED OPTICAL AND ELECTRIC CABLES OR LINES
H02H	EMERGENCY PROTECTIVE CIRCUIT ARRANGEMENTS
H02K	DYNAMO-ELECTRIC MACHINES
H02P	CONTROL OR REGULATION OF ELECTRIC MOTORS, ELECTRIC GENERATORS OR DYNAMO-ELECTRIC
	CONVERTERS; CONTROLLING TRANSFORMERS, REACTORS OR CHOKE COILS

Germany’s dominating industrial sectors include automotive, machinery and equipment, electrical and electronic, and chemical engineering. These sectors not only contribute to the national GDP, but also are the focal points of innovation of this country. Among the top ten German organizations filing the most PCT patents, at least 6 have automotive as its major or one of the major operations, including vehicle manufacturers like Continental Automotive GMBH and Audi AG, automotive components and assembly suppliers like Robert Bosch Corporation and Schaeffler Technologies AG & Co. KG, and research institutes like the Fraunhofer Society (statistics database W 2017). Germany Trade & Invest (GTAI), the economic development agency of the Federal Republic of Germany reported that internal combustion engine energy efficiency, alternative drive technologies (including electric, hybrid, and fuel cell cars), and adapting lightweight materials and electronics are the current major market trends (GTAI 2017). From electronic technologies, software solutions to metallurgy, chemical engineering, automation and drive technologies, innovation in the automotive industry drives and benefits from a number of other sectors.

In fact, these trends in the automotive sector are not limited to Germany, but Germany’s case is more noticeable and representative given its outstanding concentration of R&D, design, supply, manufacturing and assembly facilities. The automotive industry does not just source from other sectors for innovative technological support. When Enkel and Gassmann examined 25 cases of cross-industry innovation, automotive is observed as both the result and source of the original idea (Enkel and Gassmann 2010). The interactive sectors range from the ones with a closer cognitive distance like aviation and steel industry to the more distant ones like sports, medical care and games. These cases all occurred between 2005 to 2009, and indeed, the cross-industry technological interactions have become more dynamic starting from 2000, as the shuffles observed in Figs. 4, 5 and 6 of our analysis. In 2009, Germany’s Federal Ministry for Environment, Nature Conservation, Building and Nuclear Safety issued German Federal Government’s National Electromobility Development Plan (Bundesregierung 2009) specified a serial action plan to promote electromobility in Germany, which defines 2009 to 2011 for market preparation, 2011 to 2016 as market escalation and 2017 to 2020 as mass market. The first stage focuses on research and development. The Plan also identifies batteries as the weakness of Germany’s automotive sector on the path to the leading position in electromobility. The increased activities in Section G and H starting from 2000 might be a reflection of this policy. However, this is up to validation when more data covering the following years will become available.

**Robustness check:** For community tracking, we have performed the analysis under 10-year and 5-year time window settings, and found the results to be very close. The results presented in Figs. 5 and 6 are based on 5-year time windows. In addition, we have done robustness checks using the other community mapping method and with time window shifts, shown in Additional file 2: Figure S9 and Additional file 3: Figure S10 respectively using the example of B60R’s community. In Additional file 2: Figure S9, when referring to the initial year, the colored blocks layout is the same as Fig. 6 except for the colors used, which is merely due to the difference in the definition in the mapping methods. We find similar results in Additional file 3: Figure S10: There is no difference from Fig. 6 except for the 1-year shift. We have performed such robustness checks for other communities and obtained the similar results. This indicates that the community mapping method is stable and consistent in identifying central nodes and tracking communities.

**Fig. 7:**
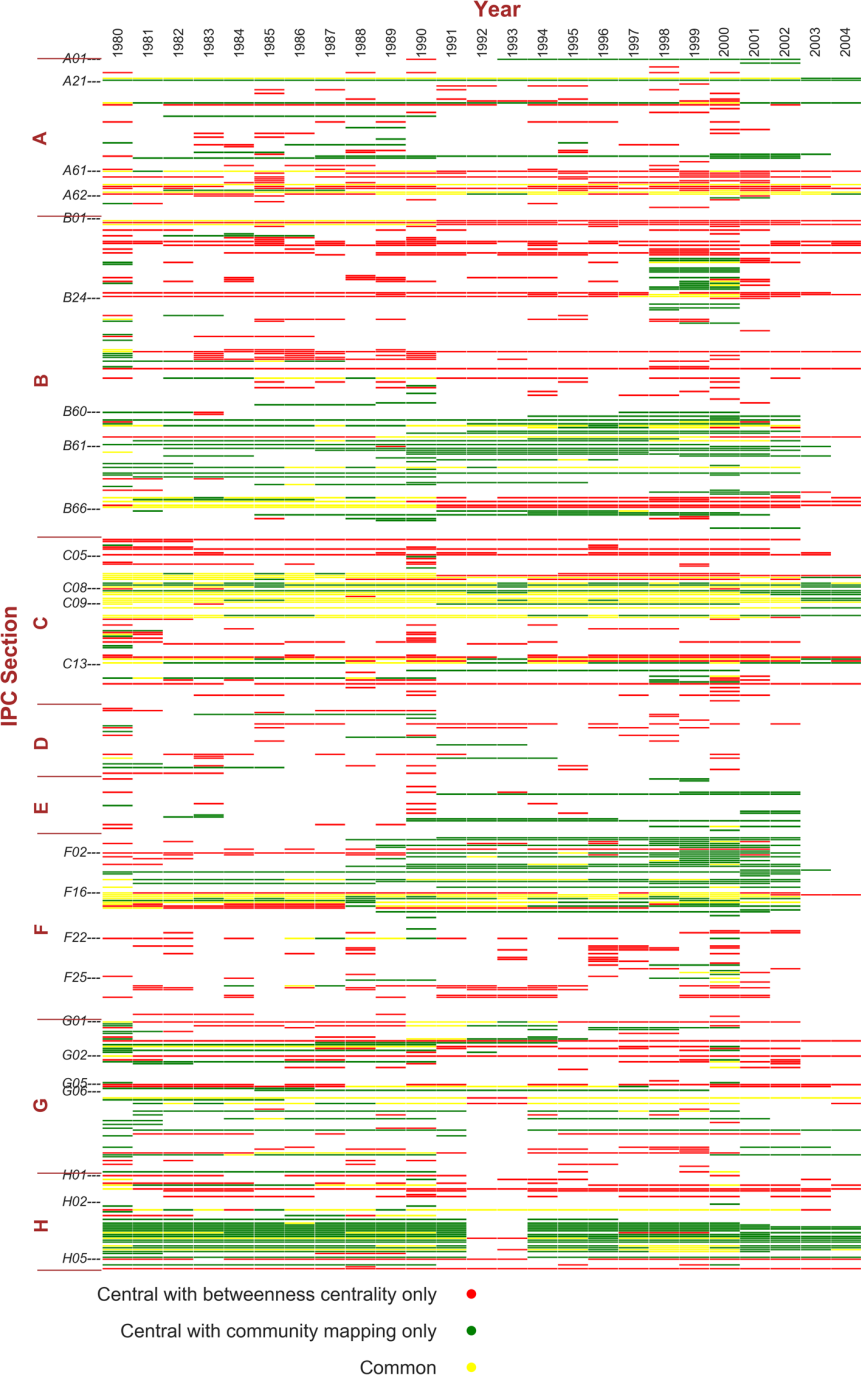
Central Nodes Distribution by Comparing the Community Mapping Method with the Betweenness Centrality Method. The x-axis indicates years from 1980 to 2004, and the y-axis indicates the nodes, i.e. IPC subclasses, ordered in IPC index. The community mapping is based on consecutive 10-year windows. Betweenness centrality values are calculated on 3-year aggregation period started with the same labeled year as the other method. Colored blocks indicate the central nodes in common and different between the two method, following the legend

**Central nodes identification methods comparison:** Alternative to the community mapping and central nodes identification method, we try to rank nodes by their betweenness centrality. Betweenness centrality is one of the most widely used measures of vertex centrality in a network (Bavelas 1948*;*Beauchamp 1965*;*Freeman 1977). Compared to other centrality measures using degree or closeness, betweenness represents the connectivity of a node as a bridge connecting two other nodes along a shortest path. We use it as an example to demonstrate the similarity and difference between our method and the conventional network centrality measures. The betweenness centrality of each node is calculated to find the nodes with the highest centrality values. In order to avoid outstanding impact from a single year, we use aggregated data from 3 consecutive years to form a network, based on which the centrality is calculated for the first of the 3 years. We present here the results comparison for the same years from 1980 to 2004. As a major difference between the two algorithms, ours provides a set of central nodes all with the same occurring rate of 1, but the betweenness centrality value of most nodes are different, ranking them from high to low. So when using the betweenness centrality method, we take the *M* nodes ranking highest by centrality values, with *M* being the size of central nodes set in the same time period using our method. For example, the central nodes set in the time windows starting with 1980 has 131 nodes, and the top 131 nodes with highest betweenness centrality rankings in the aggregated period of 1980–1982 are used for comparison. The matching rates are shown in Table 3, averaging at 32.45%. Figure 7 shows the distribution over IPC scheme using both methods. The central nodes based on our algorithm are the ones shared by both referring methods.

**Table 3:** Matching rates of central nodes by the community mapping method and the betweenness centrality method

Year	Common	Different	Match (%)
1980	53	78	40.46
1981	38	56	40.43
1982	33	60	35.48
1983	33	60	35.48
1984	32	60	34.78
1985	30	65	31.58
1986	42	54	43.75
1987	39	56	41.05
1988	36	63	36.36
1989	45	63	41.67
1990	48	79	37.80
1991	34	58	36.96
1992	24	49	32.88
1993	21	52	28.77
1994	32	67	32.32
1995	31	63	32.98
1996	22	69	24.18
1997	27	63	30.00
1998	36	80	31.03
1999	39	87	30.95
2000	53	96	35.57
2001	22	74	22.92
2002	18	59	23.38
2003	5	34	12.82
2004	6	28	17.65

The two algorithms are different by definition, and offer different information as the comparison shows. It is difficult to verify the results against ground truth, but we argue that our method has two important advantages. First, there is no arbitrary control of the number of central nodes. To study the interaction of technologies in cohesive families, to have a set of central nodes rather than a given number of top centrality nodes is intuitively closer to the real-world situation. Second, our method identifies the set of central nodes based on tracked communities over a time window, while the betweenness centrality calculated is for a single time period (3 years in the demonstrated example) - additional efforts are needed to track communities over time in order to calculate the centrality values for continuous time periods. It would only be more inaccurate to simply aggregate data in a time window of 10 years and calculate the centrality. These issues stand true for all other centrality measure. Figure 7 also shows the central nodes identified using our proposed method are more consistent and concentrated, while the top betweenness centrality nodes are more spread out over the whole IPC scheme.

Similarities between the two results also confirm the persistent and changing trends shown in Fig. 4: bio-technology in agriculture and food (A01, A21, A23), chemical technology in medical science and pharmaceutics (A61), material separation and other processing (B01), machine tools (B23), Vehicles and transport (B60-B65), organic chemistry (C7-C9), biochemistry (C12), engine technology (F1-F16), physics measuring, testing, computing and controlling (G01, G05 and G06) and electronic technology (H01-H04) are more persistent. And increasing centrality is found with B21-B23, B60-B61,C12-C13, H03-H04.

**Comparison with multislice community detection and tracking method:** To study networks that evolve over time, another methodology is to treat the changing network as slices at different points in time based on quality functions. Mucha et al. (2010) proposed a method to generalize the problem of network community structure detection using interslice coupling adjacency matrices consisting of coupling parameters between nodes in different slices. The generalized algorithm offers flexible configurations for both the resolution parameters as we used in the Louvain modularity clustering algorithm, and the interslice coupling parameter indicating connection among slices under Laplacian dynamics. This solution is applicable to the multiplex community detection task we have. To compare the results, we applied the algorithm proposed by Mucha et al. in the same 5-year time windows as shown in Figs. 6 and 7, with the same resolution set to 1.0 and the coupling parameter as 1.0. and then find the communities containing subclasses B01D and B60R, respectively. The algorithm also obtains clusters based on modularity optimization, and generates a considerable amount of very small communities. Same for the orphan nodes. Therefore, communities with 5 nodes or less are also excluded in the results for comparison, as shown in Additional file 4: Figure S11 and Additional file 5: Figure S12.

Comparing Additional file 4: Figure S11 with Fig. 5, and Additional file 5: Figure S12 with Fig. 6, obvious similarities can be observed. The “move” of B21-B30 from 1990 to 1999 is not shown for B01D. But from 1995 to 1999, most nodes in this section drop out for B06R, although they did not “move” to the community containing B01D. It verifies the marginality of this section, that they tend to have close connection to several different communities.

As mentioned before, it’s hard to determine the result of which algorithm is closer to the truth. Each method has its unique properties. The algorithm by Mucha et al. has the advantage of providing an overall picture of all the communities and their changes over time, but we have found that as the continuous time period increases, the number of clusters detected will decrease, which reduces the sensitivity to changes. When applied on shorter time period, the 2 methods have 2 steps in common: communities identification and tracking. For the first step, we argue that our method has higher stability and consistency given the Stabilized Louvain Method. Additionally, our method is capable to find the central nodes of a community, which is meaningful in the situation of this study.

**Comparison with conventional patent metrics:** Compared to the simpler, more straightforward metric used in conventional patent data analysis, the network approach is more complicated and costs more computational resources. However, we propose the network method for its advantage in studying the structure of an inter-connected system. In Gao et al. (2017), the authors showed that ranking nodes by their connections with a given “key” subclass produced different results than the network clustering method, although largely similar. In the network perspective, nodes are clustered based on their relative proximity instead of the absolute counts or frequencies. Consider the situation where a node *k* is connected to nodes in 2 clusters *A* and *B*, where *A* has more nodes than *B* and therefore gives N more occurrence/connections. A simple measure will put *k* as a key node in *A*, but the network algorithm might attribute *k* to *B* if there are other nodes in A with even stronger connections to each other.

Secondly, some structural changes may be anticipated by economic historians and policy makers as results from known actions or decisions, but they don’t usually roll out as expected, with likely differences in timing or extent. As compared to traditional methods, our approach is better suited to detect structural change and paradigmatic shifts in the technological landscape.

## Conclusion

Through the three-step procedure, we demonstrated a way to improve community detection for temporal evolving networks, and more importantly, to track the community changes over time. Using Germany as a case study, we have verified this procedure by combining industry literature and robustness checks. Methodologically, our method contributes to the literature of temporal networks analysis with a new approach. Comparisons with conventional methods have helped to prove its validity and advantages. In terms of application, it is the first of such in patent data analysis. Although the subject of interest here is technological evolution, we expect the proposed approach to become a powerful tool for studying similar systems.

## Limitations and future work

We focus our analysis on selected technological fields. Neither Fig. 4 nor Fig. 7 distinguishes the central nodes by communities. It is because the communities are not exogenously defined, and to track all the communities requires selection of a node in each community in the initial year. In fact, none of the methods discussed can show how all the communities change over time in one picture with satisfying accuracy, sensitivity and stability. Our method is more efficient in showing which nodes are the most central and investigating the evolution of the community containing certain technologies of interest.

Given that the method utilizes the information embedded in the network changes, it can be generalized for other temporal networks studies. However, the result verification still requires more work due to the reasons mentioned in the Discussion section. A next step in our research is the application in other countries or regions to expose the method to a more comprehensive check. This will also provide an opportunity to study how various factors, including policy decisions, market trends, economic growths, national or regional resources, human resources, government and business investment, would interact with technological exploration.

## Additional files

Additional file 1 Community structures of the individual sample years based on Louvain modularity optimization algorithm, with resolution of 1.0. Major communities with more than 5 nodes are in the center, with different colors indicating each unique community, surrounded by small communities with 5 nodes or less in white color. (PNG 256 kb)

Additional file 2 Tracking community B60R in consecutive 5-year time windows, mapping to the initial year. This figure differs from Fig. 6 that the overlapping community mapping reference is the initial year of each time window, using the same color coding definitions as Fig. 6. (PNG 312 kb)

Additional file 3 Tracking community B60R in consecutive 5-year time windows, starting from 1981, mapping to the previous year. This figure differs from Fig. 6 that the all the time windows are shifted 1 year forward, using the same color coding definitions as Fig. 6. (PNG 316 kb)

Additional file 4 Communities containing B01D in consecutive 5-year time windows (starting from 1980–1984) based on the multislice community detection and tracking method. Nodes in blue color are in the same community with B01D in each year. (PNG 257 kb)

Additional file 5 Communities containing B60R in consecutive 5-year time windows (starting from 1980–1984) based on the multislice community detection and tracking method. Nodes in blue color are in the same community with B60R in each year. (PNG 275 kb)
