# Supplementary figures and images for: Community evolution in patent networks: technological change and network dynamics
Source: Appl Netw Sci. 2018 Aug 13;3(1):26. doi: 10.1007/s41109-018-0090-3 (PMC6214301; doi:10.1007/s41109-018-0090-3)

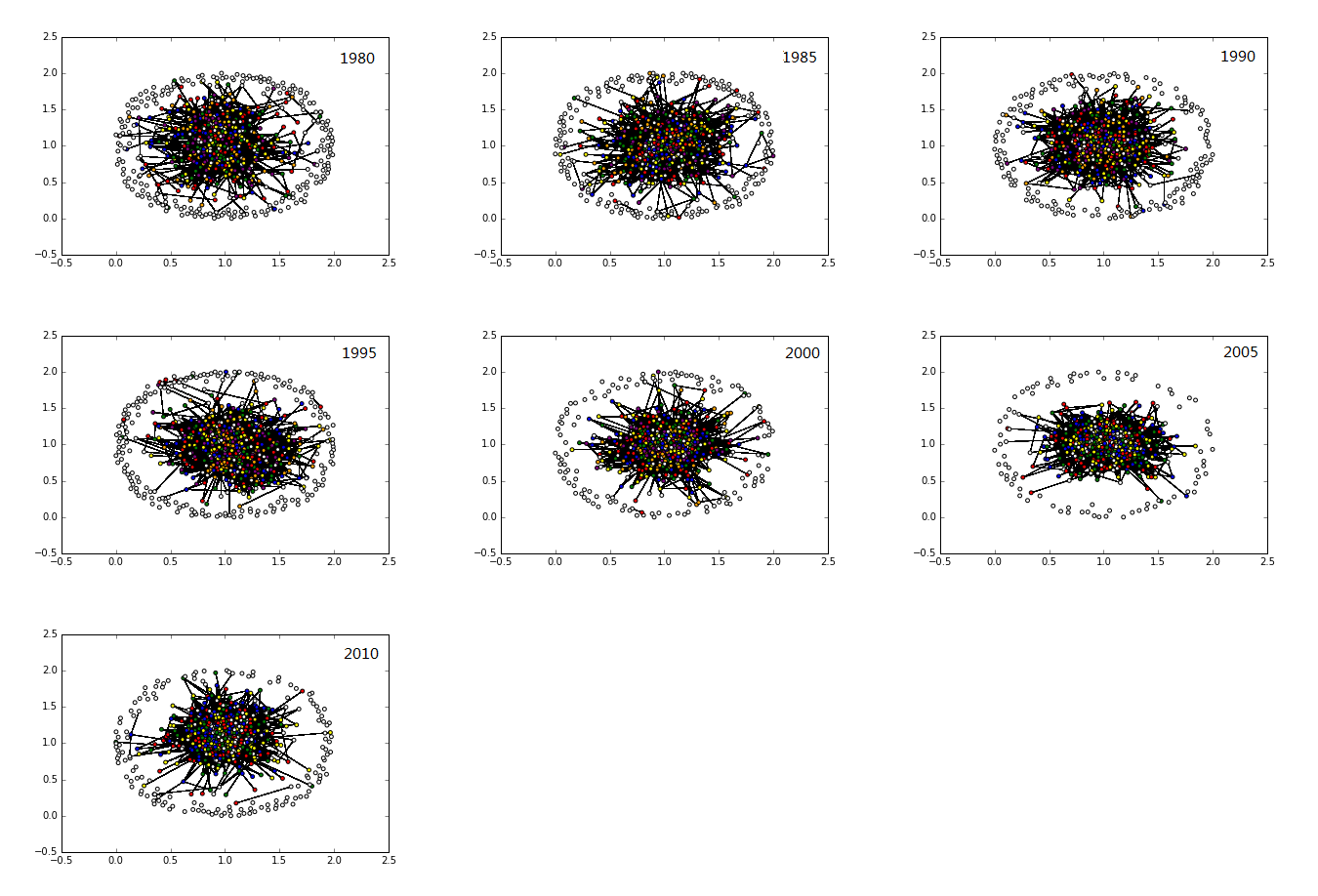

Supplement: Supplementary file 1 — Community structures of the individual sample years based on Louvain modularity optimization algorithm, with resolution of 1.0. Major communities with more than 5 nodes are in the center, with different colors indicating each unique community, surrounded by small communities with 5 nodes or less in white color. (PNG 256 kb) [file 41109_2018_90_MOESM1_ESM.png]

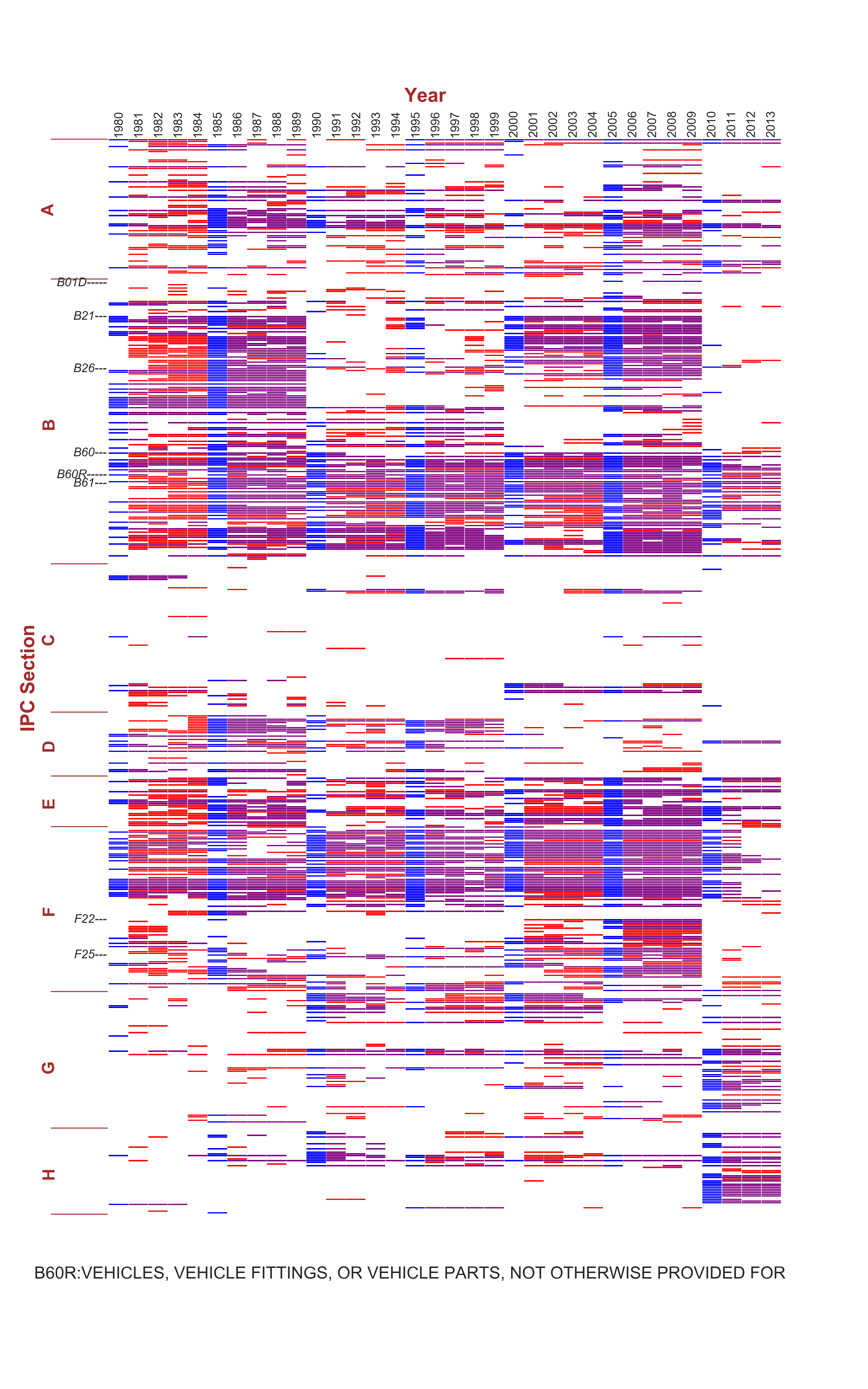

Supplement: Supplementary file 2 — Tracking community B60R in consecutive 5-year time windows, mapping to the initial year. This figure differs from Fig. 6 that the overlapping community mapping reference is the initial year of each time window, using the same color coding definitions as Fig. 6. (PNG 312 kb) [file 41109_2018_90_MOESM2_ESM.png]

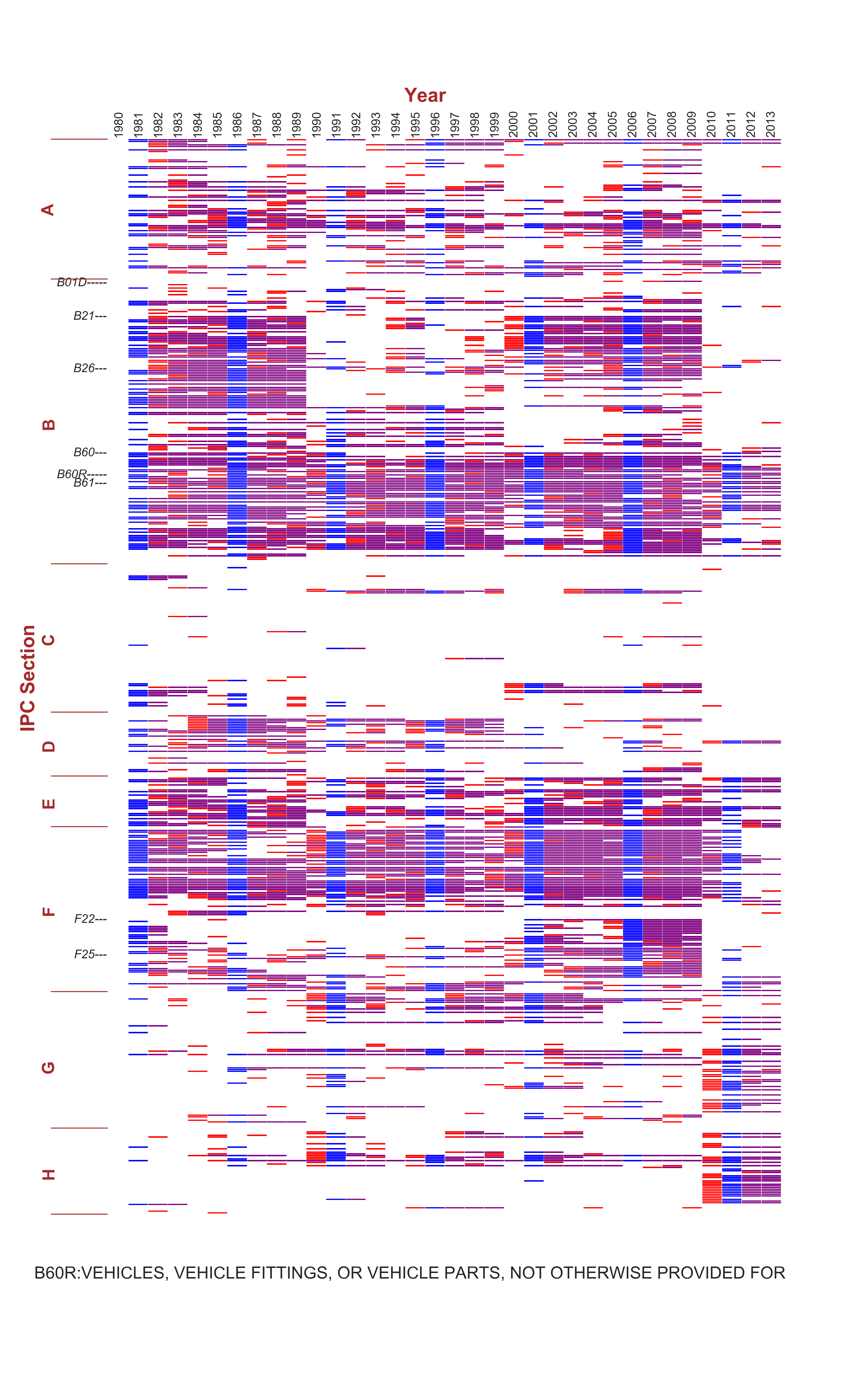

Supplement: Supplementary file 3 — Tracking community B60R in consecutive 5-year time windows, starting from 1981, mapping to the previous year. This figure differs from Fig. 6 that the all the time windows are shifted 1 year forward, using the same color coding definitions as Fig. 6. (PNG 316 kb) [file 41109_2018_90_MOESM3_ESM.png]

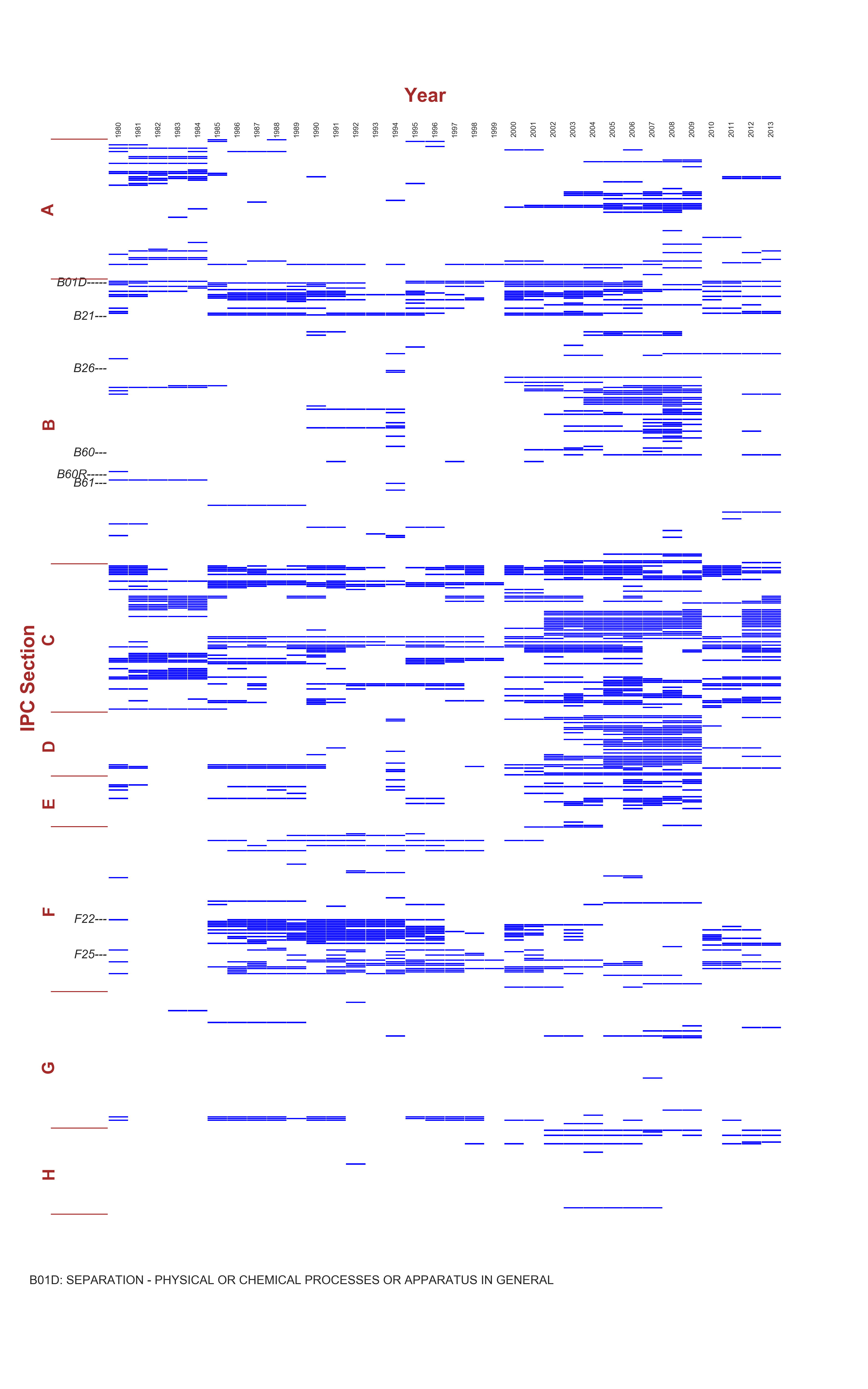

Supplement: Supplementary file 4 — Communities containing B01D in consecutive 5-year time windows (starting from 1980–1984) based on the multislice community detection and tracking method. Nodes in blue color are in the same community with B01D in each year. (PNG 257 kb) [file 41109_2018_90_MOESM4_ESM.png]

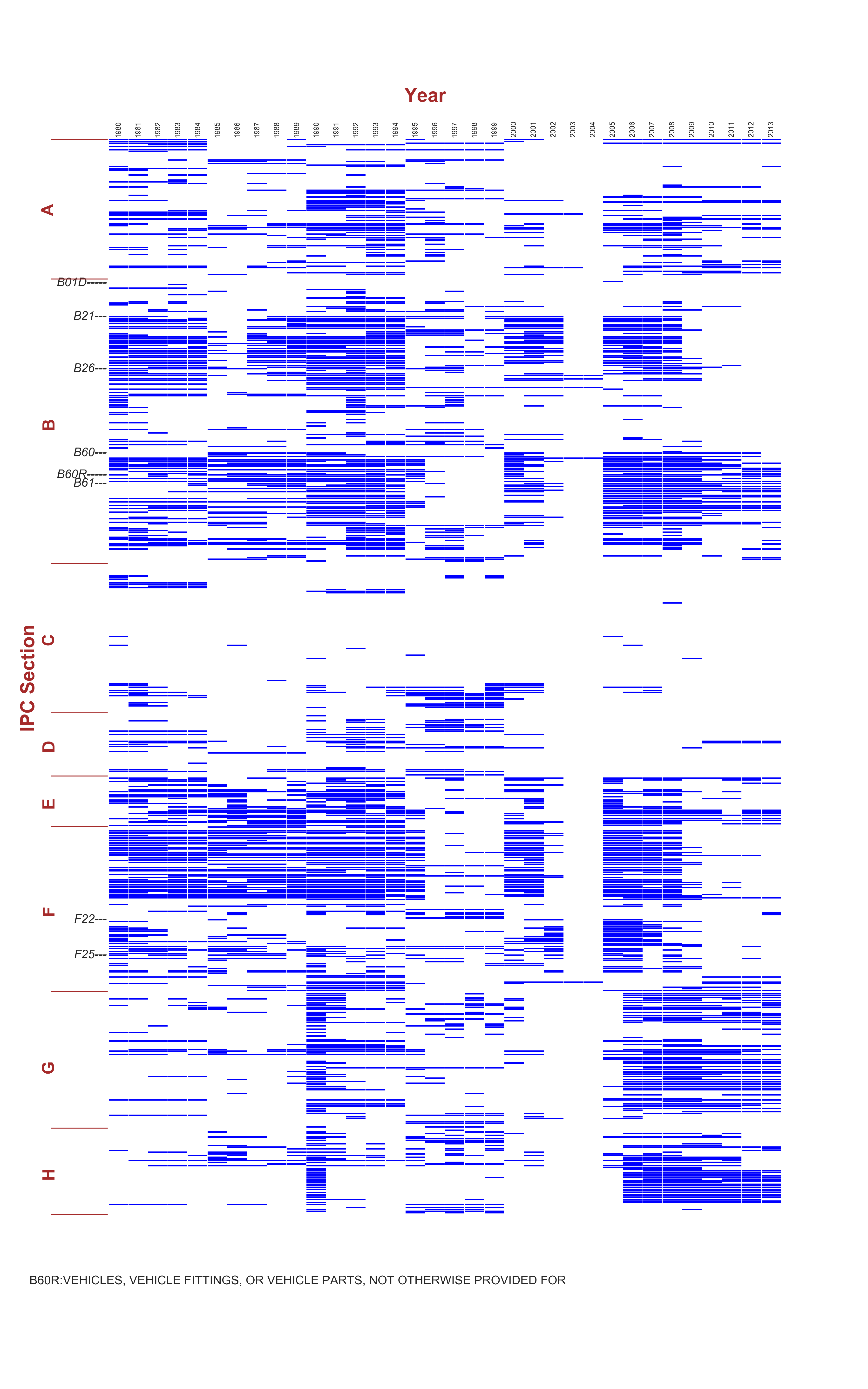

Supplement: Supplementary file 5 — Communities containing B60R in consecutive 5-year time windows (starting from 1980–1984) based on the multislice community detection and tracking method. Nodes in blue color are in the same community with B60R in each year. (PNG 275 kb) [file 41109_2018_90_MOESM5_ESM.png]
